# Book review: beyond a shadow of a diet

**DOI:** 10.1186/s40337-017-0173-z

**Published:** 2017-10-27

**Authors:** Jessica Bailes

**Affiliations:** Wesley Hospital, Ashfield, Australia

## Background

Beyond a Shadow of a Diet is an easy-to-read 338 page guidebook on how to apply the Health at Every Size ® (HAES ®) to treat binge eating in a psychoanalysis setting, and divided into three parts, 1. ‘The Problem’, 2. ‘The Treatment’ and 3. ‘The Solution’.

Part one is an excellent introduction to diet culture and its impact on binge eating, as well as on how the therapist may be complicit in the same culture. Much of this content may also be found in the book ‘Health At Every Size’ by Bacon [1].

Part two outlines the principles of Intuitive Eating as described by Evelyn Trible, and gives tips to help clients adopt the approach, as well as to overcome obstacles, and manage emotions and poor body image, all through a psychoanalysis lens. Part three returns to diet culture, and the HAES(R) principles, and gives information on how clients may pursue health in a non-diet paradigm, mainly through giving references for further reading.

The authors are two social workers, who both have over 20 years’ experience working in eating disorders using a psychoanalysis approach. The book benefits from their knowledge, many excellent examples from their extensive experience to illustrate how they have been treating binge eating disorder. It should be noted that their approach is not manualised, and therefore no studies have yet been done to evaluate the effectiveness of this approach.

## Conclusion

Overall, this book has a good brief introduction to HAES (R), but for more in-depth information on the science of HAES ®, readers may find the source material, such as the work by Linda Bacon and Evelyn Trible, more illuminating. Part two is where the true value of the book if found. There are so few books aimed at supporting the practioner in treating binge eating disorder, and this book has an in depth exploration of an alternative to Fairburn’s CBT-E [2]. This is a welcome addition to support those who would like to use HAES (R) in their treatment psychotherapy but don’t know where to start.
